# Usefulness of serum D-dimer and platelet count to mean platelet volume ratio to rule out chronic periprosthetic joint infection

**DOI:** 10.5194/jbji-7-109-2022

**Published:** 2022-05-17

**Authors:** Ernesto Muñoz-Mahamud, Eduard Tornero, José A. Estrada, Jenaro A. Fernández-Valencia, Juan C. Martínez-Pastor, Álex Soriano

**Affiliations:** 1 Department of Orthopedics and Trauma Surgery, Hospital Clinic of Barcelona, University of Barcelona, Barcelona, Spain; 2 Department of Infectious Diseases, Hospital Clinic of Barcelona, University of Barcelona, Barcelona, Spain

## Abstract

**Background**: Diagnosing periprosthetic joint infection (PJI) is challenging and usually requires the evaluation of several biomarkers. Our main aim was to evaluate the usefulness of D-dimer levels as well as the platelet count (PC) to mean platelet volume (MPV) ratio serum as biomarkers to rule out chronic knee and hip infection. **Methods**: The study enrolled a prospective cohort of 93 patients undergoing hip or knee revision. D-dimer values, PC to MPV ratio, C-reactive protein (CRP) and erythrocyte sedimentation rate (ESR) were preoperatively determined and evaluated as a predictor of PJI. The definitive diagnosis of PJI was established according to the 2018 International Consensus Meeting criteria. **Results**: A total of 24 (25.8 %) cases were postoperatively diagnosed with PJI. The median D-dimer value was significantly higher ( p < 0.001) for patients with PJI (1950 ng mL ${}^{-1}$ ) than for patients with aseptic failure (700 ng mL ${}^{-1}$ ). The area under the receiver operating characteristic curves for D-dimer, CRP and ESR was 0.820, 0.793 and 0.791 respectively. D-dimer ≥ 950 ng mL ${}^{-1}$ (91 % sensitivity, 64 % specificity), CRP ≥ 1.95 mg dL ${}^{-1}$ (61 % sensitivity, 90 % specificity) and ESR > 20 (74 % sensitivity, 82 % specificity) were identified as the values with the best balance between sensitivity and specificity. The mean PC to MPV ratio was 37.0 for PJI patients and 29.8 for patients in the aseptic revision cohort ( p=0 .067). **Conclusions**: Serum D-dimer levels appear very unlikely to remain normal in the presence of chronic PJI. The 91 % sensitivity when considering 950 ng mL ${}^{-1}$ as the threshold highlights D-dimer as the most accurate initial test to rule out chronic PJI. Conversely, the PC to MPV ratio may be of limited value for accurately diagnosing PJI.

## 1 Introduction

Despite periprosthetic joint infection (PJI) being one of the most devastating complications after total joint arthroplasty (TJA), its diagnosis still remains challenging (Deirmengian et al., 2014; Parvizi and Della Valle, 2010). An updated denotation of the minor criteria scoring system for diagnosing PJI was latterly depicted by the 2018 International Consensus Meeting (ICM) on Musculoskeletal Infection (Parvizi et al., 2018). However, many of these criteria (for instance, cultures or synovial fluid parameters) are available only after invasive procedures or even after prosthetic revision surgery.

Although several serum biomarkers (such as C-reactive protein (CRP) level, erythrocyte sedimentation rate (ESR) or D-dimer) were included as minor criteria in ICM guidelines for PJI (Parvizi et al., 2018), most of these biomarkers may not be altered in those infections caused by low-virulence microorganisms (Fernandez-Sampedro et al., 2015; Staats et al., 2017; Vargas-Reverón et al., 2020). Conversely, some studies have suggested that serum D-dimer levels may be increased in cases of chronic infection (Shahi et al., 2017; Hu et al., 2020; Pannu et al., 2020a, b). Even though the 2018 ICM minor criteria have been recently validated (Abdelaziz et al., 2020), some controversy still remains regarding the usefulness of D-dimer for diagnosing chronic PJI (Xu et al., 2019; Li et al., 2019; Hao et al., 2020). Subsequently, the measurement of the platelet count (PC) to mean platelet volume (MPV) ratio has been proposed as a possible predictor of PJI; yet its validation is still uncertain.

The aim of the present study was to evaluate the usefulness of both serum D-dimer levels and the PC to MPV ratio as preoperative biomarkers for chronic PJI in cases of knee and hip revision.

## 2 Materials and methods

This single-center prospective study enrolled consecutive cases of hip and knee arthroplasty revisions, performed at our institution between January 2018 and February 2019. For the present study, only patients with preoperative diagnosis of aseptic loosening or chronic PJI were included. Patients who underwent arthroplasty revision due to periprosthetic fracture or dislocation and patients with early PJI who underwent open debridement with implant retention (DAIR) and the second stage of a two-stage septic revision were excluded from the study. The Institutional Review Board approved the study (register number: HCB/2019/0827).

In all cases, the preoperative study started with a comprehensive physical examination and plain x-rays. Preoperative quantification of CRP and ESR was routinely performed. To test whether any of these tests showed suspicion of infection, synovial fluid was aspirated (in hip cases the fluid was obtained by percutaneous puncture guided by computerized tomography) and submitted for cultures (as well as for white blood cell count only in those cases in which enough volume was obtained). In addition, venous blood samples were collected by nurses on the day of admission and analyzed for serum D-dimer levels, only for study purposes. D-dimer measurement was performed using Innovance^®^ (Siemens Healthcare Diagnostics Products GmbH, Germany), a particle-enhanced immunoturbidimetric assay for the quantitative determination of cross-linked fibrin degradation products: the reagent contains polystyrene particles coated with monoclonal antibodies with specificity against D-dimer. In the presence of D-dimer in the sample, an Ag–Ac union is produced in the form of aggregates that produce a change in the turbidity of the sample that can be quantified and extrapolated to a given D-dimer concentration.

Additionally, both PC and MPV were preoperatively measured in all cases. Data recording featured demographics (age and sex), the need for anticoagulant therapy, the reason for revision surgery, implicated joint (hip or knee), the presence of fistula, microbiological results, synovial fluid laboratory findings, anatomopathology of periprosthetic tissue and baseline preoperative serum analysis including CRP (mg dL ${}^{-1}$ ), ESR (mm h ${}^{-1}$ ), D-dimer (ng mL ${}^{-1}$ ) and PC to MPV ratio. All surgical interventions were done by surgeons specifically specialized in revision arthroplasties in a laminar airflow equipped operating theater. Antibiotic prophylaxis was routinely administered prior to the beginning of the surgical procedure: the standard intravenous antibiotic prophylaxis protocol consisted of 2 g of ceftazidime IV and 800 mg of teicoplanin IV along the induction of anesthesia. A regimen consisting of 2 g/8 h of ceftazidime IV and 1 g/12 h of vancomycin IV was withheld until the obtention of definitive cultures. In all cases, synovial fluid was aspirated and sent to laboratory for white blood cell count, neutrophil count and microbiological analysis. Our microbiological protocol for culture sample collection features two synovial fluid (routinely inoculated into blood culture flasks) as well as two tissue samples from the neo-synovium, plus two tissue samples from the interface membrane. In addition, two interface membrane samples were submitted for histology regarding the adapted by Feldman and Mirra's criteria (Mirra et al., 1976; Feldman et al., 1995). The definitive diagnosis of PJI was established according to the criteria defined at the 2018 ICM on Musculoskeletal Infection (Parvizi et al., 2018).

Continuous variables were displayed as mean or median and standard deviation (SD) or interquartile range (IQR) and subsequently analyzed using Student's t test or the Mann–Whitney U test depending on the Kolmogorov–Smirnov test of normality. Continuous variables were categorized as well according to mean value (age < 72 and ≥ 72 years) or according to data obtained in the receiver operating characteristic (ROC) curve (D-dimer < 950 ng and ≥ 950 ng mL ${}^{-1}$ ; CRP < 1.95 and ≥ 1.95 mg dL ${}^{-1}$ ; ESR ≤ 20 and > 20 mm h ${}^{-1}$ ). Qualitative variables were described by absolute frequencies and percentages and were compared using the chi-squared test or Fisher's exact test when necessary. The predictive values of D-dimer, CRP, ESR and PC to MPV ratio were checked for correctly indicating the presence of PJI via a receiver operating characteristic curve. Values with the best balance of sensitivity and specificity and values corresponding to 90 % sensitivity and 90 % sensitivity were calculated for all curves. Statistical significance was defined as a two-tailed p < 0.05. The statistical analysis was performed with SPSS v. 20.0 (IBM Corp., Armonk, NY, USA).

## 3 Results

A total of 93 cases were included in the study. The mean (SD) age of the cohort was 71.4 (9.0) years, and 59 (63.4 %) were female. The involved joint of the prosthetic joint replacement was the knee in 66 (71.0 %) cases and the hip in 27 (29.0 %) cases. The preoperative diagnosis was aseptic loosening in 70 (75.3 %) cases and PJI in 23 (24.7 %) cases (17 cases underwent one-stage revision and 6 cases underwent two-stage revision). Only six (6.5 %) patients received anticoagulant therapy prior to surgery. According to the criteria proposed at the 2018 ICM on Musculoskeletal Infection (Parvizi et al., 2018), a total of 24 (25.8 %) cases were postoperatively diagnosed with PJI. The infection was polymicrobial in 3 (13.0 %) cases, and the most frequently isolated microorganisms were: coagulase-negative staphylococci (12 cases, 52.2 %), *Staphylococcus aureus* (2 cases, 8.7 %), *Staphylococcus haemolyticus* (1 case, 4.3 %), *Propionibacterium acnes* (1 case, 4.3 %) and *Proteus mirabilis* (1 case, 3.4 %). In 3 cases (13.0 %), no microorganism was isolated in cultures obtained during surgery.

**Figure 1:**
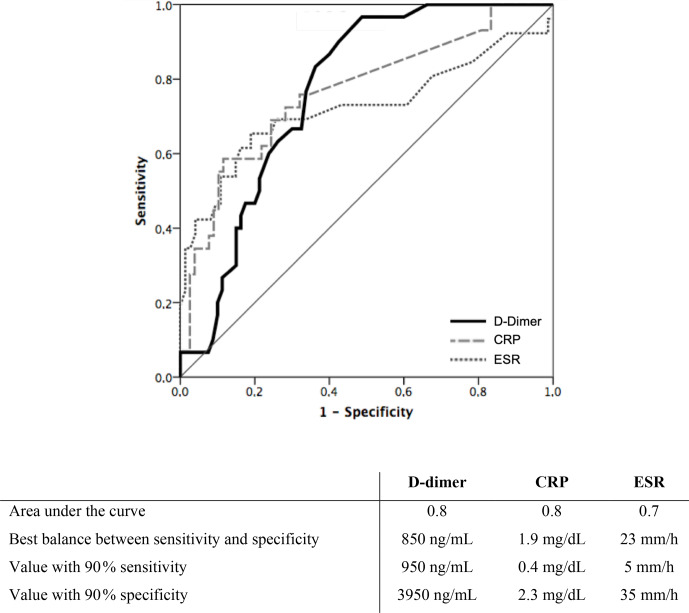
Receiver operating characteristic curve of serum biomarkers (D-dimer, CRP and ESR) for preoperative diagnosis of prosthetic joint infection featured in the 2018 International Consensus Meeting criteria of prosthetic joint infection.

The median (IQR) D-dimer concentration prior to surgery was 900 (450–1950) ng mL ${}^{-1}$ . CRP values were available in 92 (98.9 %) cases, and median (IQR) concentration was 0.40 (0–1.85) mg dL ${}^{-1}$ . ESR values were available in 90 (96.8 %) cases, and median (IQR) sedimentation rate was 16 (7–28) mm h ${}^{-1}$ . Values of pre-operative D-dimer, CRP and ESR were carefully evaluated as a predictor of PJI. The mean PC to MPV ratio was 37.0 for PJI patients and 29.8 for patients in the aseptic revision cohort ( p=0 .067). The area under the ROC curve was 0.592 (95 % CI 0.443–0.742, p=0 .187). A ratio > 41 was identified as the value with the best balance between sensitivity (39 %) and specificity (87 %). Figure 1 shows the D-dimer, CRP and ESR receiver operating characteristic curves. The areas under the receiver operating characteristic curves for D-dimer, CRP and ESR were 0.820 (95 % CI 0.733–0.907), 0.793 (95 % CI 0.686–0.899) and 0.791 (95 % CI 0.664–0.918), respectively. D-dimer ≥ 950 ng mL ${}^{-1}$ (91 % sensitivity, 64 % specificity), CRP ≥ 1.95 mg dL ${}^{-1}$ (61 % sensitivity, 90 % specificity) and ESR ≥ 20 (74 % sensitivity, 82 % specificity) were identified as the values with the best balance between sensitivity and specificity. Values of D-dimer, CRP and ESR corresponding to 90 % sensitivity and 90 % specificity are shown in Fig. 1. Median (IQR) D-dimer, CRP, and ESR, according to the 2018 ICM diagnosis criteria of PJI, are shown in Table A1.

The positive (PPV) and negative predictive value (NPV) for all cutoff points studied are summarized in Table A2. Best NPV were obtained when D-dimer ≥ 950 ng mL ${}^{-1}$ (NPV = 96 %), whereas best PPV were obtained when CRP ≥ 1.95 mg dL ${}^{-1}$ (PPV = 68 %). Combination of both cutoff points (D-dimer ≥ 950 ng mL ${}^{-1}$ and CRP ≥ 1.95 mg dL ${}^{-1}$ ) only increased the PPV (PPV = 78 %).

## 4 Discussion

Recent literature proposes different coagulation factors to diagnose PJI (Xu et al., 2019; Li et al., 2019; Hao et al., 2020). Serum D-dimer values are widely used for the monitoring of fluctuations in fibrinolytic activity in reference to the presence of deep venous thrombosis (Hansrani et al., 2017; Borgen et al., 2013). However, noticeable evidence has recently been elucidated that hints at an elevation of serum D-dimer levels in the presence of systemic inflammation or infection, primarily regarding the joint (Gris et al., 2011; Schwameis et al., 2015). It has been suggested that, as in the presence of persistent PJI, an inflamed synovium secretes a high amount of both inflammatory factors and fibrin. Subsequently, their degradation may result in an increased concentration of both synovial fluid and serum D-dimer levels, making its simple measurement widely available and extensive (Busso and Hamilton, 2002). Even so, while Ribera et al. (2011) reported high concentrations of synovial fluid D-dimer in foals with septic arthritis (Ribera et al., 2011), the real influence of PJI on serum D-dimer levels remains controversial.

Shahi et al. (2017) conducted a prospective study including 245 patients undergoing both primary and revision arthroplasty in which, as in the present study, the median D-dimer serum values were significantly higher for those patients with PJI. They reported higher median values for both aseptic (299 ng mL ${}^{-1}$ ) and septic cases (1110 ng mL ${}^{-1}$ ) compared to our results (800 and 1950 ng mL ${}^{-1}$ respectively). They determined 850 ng mL ${}^{-1}$ as the ideal threshold for the diagnosis of PJI and reported 89 % sensitivity and 93 % specificity. In the present study, D-dimer ≥ 950 ng mL ${}^{-1}$ was identified as the value with the best balance between sensitivity (91 %) and specificity (64 %). Whereas this value resembles the threshold reported by Shahi et al. (2017), we report a significant lower specificity. On the contrary, our reported sensitivity is significantly ( p<0 .001) higher with D-dimer (91 %) than with CRP (61 %).

According to the most recent data, the evidence supporting the use of serum D-dimer for the diagnosis of periprosthetic joint infection is still scarce and contradicting. Pannu et al. (2020a) have recently reported a high sensitivity (95.9 %) and NPV (90.9) but low specificity (32.3 %), which remains in concordance with our presented data. Accordingly, Hu et al. (2020) also described a significantly higher mean D-dimer level in patients with PJI, reporting 87.5 % sensitivity (Hu et al., 2020). However, like Shahi et al. (2017), they also report a high specificity (89.2 %). On the other hand, other authors state that D-dimer measurement entails very limited diagnostic values for PJI (Xu et al., 2019; Li et al., 2019; Hao et al., 2020), with others even suggesting that alternative plasma fibrinolytic markers may perform better than D-dimer, for instance plasma fibrinogen (Li et al., 2019; Hao et al., 2020) and both platelet count and mean platelet volume (Paziuk et al., 2020).

In spite of the recent literature, there is still contradicting evidence supporting the use of serum D-dimer to diagnose PJI in revision THA and TKA. A recent meta-analysis by Wang et al. (2022) analyzes a total of 10 studies in which the combined sensitivity of D-dimer in diagnosing periprosthetic infections is 0.81 and the combined specificity is 0.74. However, out of the 10 featured studies, only 4 of them were prospectively designed, and only 2 studies specifically studied chronic cases. The meta-analysis elucidates a significant heterogenicity in regards to different sampling types and laboratory detection methods.

There is still some controversy in reference to the most accurate threshold to rule out PJI. In the meta-analysis by Wang et al. (2022), a high variety of thresholds have been reported, ranging from 756 to 1250 ng mL ${}^{-1}$ . In all, it seems that D-dimer values under 850 ng mL ${}^{-1}$ almost discard the presence of PJI (Shahi et al., 2017; Pannu et al., 2020b; Abdelaziz et al., 2020). In the present study, D-dimer ≥ 950 ng mL ${}^{-1}$ was identified as the threshold with the best balance between sensitivity and specificity. D-dimer values may be elevated even in the absence of infection, but their levels seem very unlikely to remain unaltered in the presence of chronic PJI. Thus, D-dimer should always be considered at the initial screening of PJI. Therefore, when a low-virulence microorganism is under suspicion and conventional biomarkers (CRP and ESR) remain normal, increased serum D-dimer levels should warn clinicians about a possible misdiagnosed PJI.

In addition, recent data elucidate the role of platelets in the innate response of the human body to chronic infection and inflammation. Indeed, it has been stated that platelets may feature antimicrobial properties, aiding the immune system response to infection (Shahi et al., 2017). Accordingly, Paziuk et al. (2020) elucidated an association between PJI and platelets by describing a PC to MPV ratio of 33.4 for PJI cases compared to 25.7 for aseptic cases (Paziuk et al., 2020). However, the PC to MPV ratio obtained from the present study (37.0 for PJI cases and 29.8 for aseptic cases) did not show significant differences regarding the septic and the aseptic cohort. In addition, the low sensitivity at its best balance between sensitivity (39 %) and specificity (87 %) poses an important limitation as an aid to rule out chronic PJI. However, the high specificity may be helpful so as to confirm the absence of infection.

Our findings present several limitations. Firstly, patients under treatment with anticoagulant therapy, immunosuppressive therapies and/or with systemic inflammatory diseases were not excluded from the study. However, including patients with different conditions provides a more realistic cohort so as to assess D-dimer usefulness in the clinical setting. Secondly, a major drawback is elucidated in reference with the limited number of cases included in the analysis. This limitation arises as the aftermath of a single-center project regarding a low prevalent pathology; this unmissable limitation may explain the differences between our results and previously reported literature. Forthcoming larger prospective randomized studies are expected so as to substantiate our observations.

## 5 Conclusions

We conclude that serum D-dimer assessment should always be considered as a useful test to rule out chronic PJI, especially in those cases caused by low-virulence microorganisms in which conventional tests may lead to misdiagnose. On the contrary, the PC to MPV ratio may be of limited value for accurately diagnosing PJI and should be studied further.

## Supplement

10.5194/jbji-7-109-2022-supplement The supplement related to this article is available online at: https://doi.org/10.5194/jbji-7-109-2022-supplement.

## Data Availability

All research data can be accessed in the anonymized database in the Supplement. For access to raw data, contact the corresponding author (Ernesto Muñoz-Mahamud: e.munoz.mahamud@gmail.com).

## Appendix

The supplement related to this article is available online at: https://doi.org/10.5194/jbji-7-109-2022-supplement.
