# Supplementary material for: Usefulness of serum D-dimer and platelet count to mean platelet volume ratio to rule out chronic periprosthetic joint infection
Source: J Bone Jt Infect. 2022 May 17;7(3):109–15. doi: 10.5194/jbji-7-109-2022 (PMC9128364; doi:10.5194/jbji-7-109-2022)
Supplement: The supplement related to this article is available online at: https://doi.org/10.5194/jbji-7-109-2022-supplement. [file jbji-7-109-supplement.zip › jbji-7-109-2022-supplement-title-page.pdf]

Supplement of J. Bone Joint Infect., 7, 109–115, 2022
https://doi.org/10.5194/jbji-7-109-2022-supplement
© Author(s) 2022. CC BY 4.0 License.

JBJI
Journal of Bone and Joint Infection
Open Access

Supplement of

Usefulness of serum D-dimer and platelet count to mean platelet volume ratio to rule out chronic periprosthetic joint infection

Ernesto Muñoz-Mahamud et al.

Correspondence to: Ernesto Muñoz-Mahamud (e.munoz.mahamud@gmail.com)

- jbji-7-109-2022-supplement-title-page.pdf
- Data D-Dimer.sav
- Data D-Dimer.xlsx

The copyright of individual parts of the supplement might differ from the article licence.
